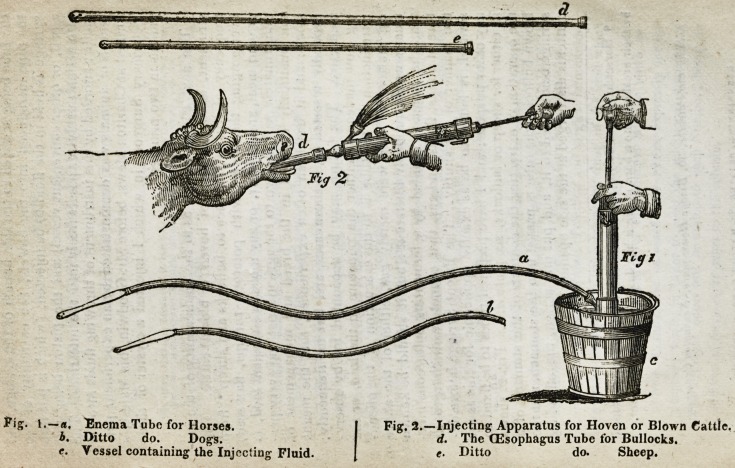# A Vindication of Read's Patent Syringe

**Published:** 1826-06

**Authors:** 


### VINDICATION

OF

### READ'S

### Patent Syringe,

*Against interested opposition and unphilosophical objections,*

WITH

### PROFESSIONAL TESTIMONIALS

OF ITS

### SUPERIOR UTILITY,

AND

***Directions by which its employment is rendered easy and certain.***

---

**BY JOHN READ,**

**Maker to the Army, and the Honorable East India Company's Forces; Inventor of the Veterinary Syringe for removing Intestinal Obstruction of Horses, and Sporting Dogs; and for relieving Blown Cattle, &c. &c.**

---

" Probatum est."

---

LONDON:

Printed by W. Glendinning, 25, Hatton Garden.

No. 230. 32

### A VINDICATION

OF

### READ'S PATENT SYRINGE,

&c. &c.

---

In the month of August, 1820, I took out a Patent for a Surgical Syringe, which has since claimed the attention of the Profession and the Public, not only in this kingdom, but of Foreign States also. The principal uses of this Instrument are, for administering Enemas, and for extracting poisons from the stomach; although it is subordinate to several other valuable purposes which I shall hereafter enumerate.

The Cylinder of the Syringe is about 7 inches in length, and ¾ of an inch in diameter; the extremity contracted and perforated by an opening considerably smaller than the internal diameter of the body of the Instrument; within this opening is a chamber containing a spherical valve, which, by rising into the upper part of the chamber, where a vacuum is formed by elevating the piston, admits the fluid to pass freely into the Syringe, but as soon as the piston is depressed, the contents of the Syringe presses the valve close upon the aperture, and prevents its escape through the opening by which it was received.

To give exit to the contents of the Syringe, a side branch is constructed, furnished with a valved chamber, similar to the one above described, but so placed as to act in direct opposition to it; so that when the Syringe has been filled from the extremity, and pressure is made by depressing the piston, the fluid closes the lower valve, and opens the lateral one, and consequently escapes through the latter aperture. To facilitate the operation of the instrument, a small pipe communicates with the upper extremity of the Syringe, which gives free ingress and egress to the atmosphere during the action of the piston, a circumstance essentially necessary in causing the instrument to work easily and perfectly.

The other part of the apparatus consists of a tube 28 inches in length, to be introduced into the stomach in removing poisons; an elastic tube 18 inches in length (with ivory pipes) for administering enemas; and a guard for the protection of the œsophagus tube in operations upon the stomach.

The contents of the case containing the enema apparatus consists of the following:

1st—The Syringe, or Pump.
2nd—The Enema Tube, armed with a brass socket at one extremity, and a screw at the other.
3rd—Three Ivory Pipes and Sockets; the small pipe for children, and the curved pipe for self injection; that which is perforated with small holes being used only in female complaints. The bulb socket is used only in *self* administration of enemas, and in *all other* cases the *flat* socket.

### Directions for Administering Enemas.

Screw the flexible tube to the side branch of the Syringe, and insert either the flat or the bulbed ivory socket as the case may require, (as explained before) into the opposite extremity, and, lastly, screw into the ivory socket, the proper pipe. The invalid, if in bed, should lay upon one side, as near the edge as convenient, and the pipe being introduced under the bed clothes, either by the patient or an assistant, is passed gently into the bowel. A basin containing the enema is next brought to the bed side, and the extremity of the Syringe being inserted into it, the assistant slowly pumps the fluid into the bowels. As a security against any of the fluid escaping during the operation, the patient may press a soft napkin to the part, which serves also to prevent a displacement of the pipe.

For self injection the patient may sit upon a chair, and place the vessel containing the enema, upon another chair before him, and in this manner, without any assistance, the instrument may be used with the greatest ease and facility. This is shewn by *fig.* 1, of the plate at page 7.—*a.* the enema tube—*c.* the basin—*d.* the curved pipe.

For much interesting matter respecting the utility of enemas, (especially the domestic practice of it) I respectfully beg leave to refer the reader to a valuable little sketch of this subject lately published by Mr. Scott.

At the suggestion of some medical gentlemen, I have provided several detached parts to be used with the Syringe, for some particular parts of medical practice which I shall here briefly enumerate.

### Injecting the Bladder.

In cases of retention of urine, it frequently happens that in consequence of hæmorrhage and other causes, the catheter becomes so obstructed that the bladder cannot be emptied: It was suggested to Mr. Scott by Dr. Cloquet, a celebrated Surgeon of Paris, to effect this purpose by fixing a pump to the catheter. The Patent Syringe performs this operation with extreme facility, and has been honoured with the entire approbation of Dr. Cloquet. For injecting the bladder, which is an operation every day becoming more frequent, it is of course equally eligible. For these purposes I have constructed elastic gum catheters to be fixed to the extremity of the enema tube—*see* e *in the following plate.*

As an Apparatus for conveying nourishment into the stomach of Persons afflicted with Stricture of the Œsophagus, the Patent Syringe is found to possess obvious advantages.*

---

### Cupping and Drawing the Breasts.

This Pump is also capable of being adjusted to cupping-glasses, by which any degree of exhaustion can be made that the operator desires; and in the same manner it may be rendered a very effectual Instrument for drawing the breasts of puerperal females. I have had glasses made for these uses, which may be obtained with the rest of the Apparatus—*see* **f. g.** *in the succeeding plate.*

### Tobacco Fumigation.

For the purpose of introducing the smoke of Tobacco into

---

* Stimulating liquids ought also to be carefully thrown into the stomach of persons under suspended animation from drowning, &c.

the intestines, I have fitted a Canister to the Syringe, by which the operation is performed with more certainty and ease than with the old medical apparatus. It is used in the following manner:

Unscrew the cap of the canister, and take out the perforated plunger; put in the tobacco (half an ounce or an ounce) and replace the plunger lightly upon it; then put on the cap and screw it to the end of the Syringe; hold a lighted candle close under the bottom of the canister, and a stroke or two of the piston of the Syringe will light the tobacco. The enema tube being now fixed to the side branch, and the pipe introduced into the rectum, the tobacco smoke is forced into the intestines, as shown by *fig.* 2, in the following plate.

*Fig.* 1. *Fig.* 2.

THE OPERATION OF EVACUATING POISONS FROM THE STOMACH.

*Fig.* 2. *Fig.* 1.

With the addition of the Œsophagus tube and the guard, the case described at page 4, contains all that is necessary for withdrawing the contents of the stomach, the operation for which, is represented above.

*Injecting the Stomach.* The Enema tube is first screwed to the lateral branch of the Syringe, and the Œsophagus tube being passed into the stomach, the brass joint at its extremity is now inserted into the socket at the end of the Enema tube. The fluid having been put into a basin, the end of the Syringe is to be immersed in it, and the piston being put into action, any quantity may be thrown into the stomach that may be desired—*see fig.* 1. *a.* the Enema tube.—*b.* the Œsophagus tube.—*c.* the basin.

*Emptying the Stomach.* A sufficient quantity of fluid having been injected into the stomach by the above process, the Enema tube is to be withdrawn from the Œsophagus tube (without removing the former from the Syringe, or the latter from the stomach) and the joint of the œsophagus tube inserted into the extremity of the Syringe; let an assistant now hold a vessel to the end of the enema tube, and by working the piston, the contents of the stomach may speedily be pumped into it, as is shewn in figure 2, of the drawing. By thus transferring the end of the œsophagus tube from one situation to the other, the two processes of washing and emptying the stomach may be repeated as often as is judged necessary by the operator. Thus it is seen that the Syringe is furnished with two valvular apertures, through *one of which* the contents of the stomach passes into the cylinder, and are then immediately forced through *the other*, into the receiving vessel. This double operation is effected by repeated strokes of the piston, which slides so easily, that an infant may use it.—In withdrawing the contents of the stomach, the lateral branch of the Syringe should be turned upwards towards the patient's face, and the instrument may be held a little obliquely, which preserves the valves upon their proper bearings.—*see fig.* 2.—*a.* the Enema tube—*b.* the Œsophagus tube—*c.* the basin.

*The New Method of Operating with Read's Patent Syringe*

An improved mode of removing poison from the stomach with the instrument which I have had the honor of introducing to public notice, was first adopted in Saint Thomas's Hospital, and has since been performed successfully in a variety of cases, as represented in the following sketch.

*a.*—Guard, to be introduced between the teeth, for protecting the Œsophagus tube from injury.

This is by far the quickest, easiest, and most simple mode of operating that has hitherto been devised, requiring no shifting of the apparatus, nor interruptions of the operation. It consists simply in filling the stomach, (according to the method of fig. 1, in the preceding plate,) until surcharged, or until it begins to re-act upon its contents, when the fluid regurgitates by the mouth. The pumping being now continued, the contents of the stomach are washed up, and forced, by the power of the pump, through the œsophagus (by *the side* of the tube,) into a vessel held under the chin to receive it. The operation may be continued as long as the Surgeon thinks proper, or until the fluid returns unchanged, which indicates the thoroughly cleansed state of the stomach. The operator may occasionally supend the action for an instant, if necessary, to allow the patient to inspire. By this means the fluid may be injected in the quantity of three quarts a minute.

As an Apparatus for conveying nourishment into the stomach of Persons afflicted with Stricture of the Œsophagus, the Patent Syringe is found to possess obvious advantages.

I shall now make a few brief remarks in vindication of my instrument against an ungenerous opposition which has been offered to it by certain interested persons, who have been maliciously active in depreciating its merits by false insinuations, for the mere purpose of selling instruments of their own manufacture. First, the spherical metallic valve (which belongs exclusively to my Syringe) they say is inferior to spiral and leather valves. Let me answer this by asking, is there any figure in geometry so simple as a sphere, or liable to so little destructive friction? Again, is not a spiral valve or any other that is kept to its bearings by a spring, liable to speedy and certain derangement by the oxidizement occasioned by alternations of moisture and dryness? And further, does not the natural hygrometrical property of animal substances which subjects them to a wide range of expansion and contraction, render these substances highly unfit for the nicety of valvular coaptation, and do they not by the chemical agency of heat and moisture, undergo an alteration in structure that proves destructive to their use? Secondly, my adversaries assert, that the valves of my Syringe become choked in those cases where it is necessary to inject a glutinous fluid, and if laid by without cleaning after such an operation, would be found clogged up when subsequently wanted. The first part of this assertion is not only a base falsehood, a gratuitous lie coined to pass current with other forgeries with which they endeavour to ruin the merit of simplicity, but it is an actual absurdity in experimental philosophy, and its fabricators, had they known any thing themselves of hydrostatics, never could have expected to impose a belief, even on the most credulous, that *a fluid*, though it were as tenacious as bird lime, could resist a pressure upon its surface (with a vacuum behind it) equal to 15 pounds to a square inch. But this calumnious assertion has been disproved by reiterated cases in which glutinous fluids have been injected, and I defy my adversaries to produce a single instance of what they advance. I boldly assert there is no fluid either natural or artificial, that can obstruct the valves of my Syringe; and I will engage to empty the stomach of an Alderman, though he may have dined upon turtle-soup with macaroni, and washed it down with mucilage of tragacanth or quince seeds. But the cream of the above charge is, that the valves will be found clogged if they are not cleaned after glutinous fluids have been used. Cunning Rogues!! Pray, Mr. Wiseacres, do you ever dissuade a customer from buying a lancet because it is subject to become rusted and "*clogged*" if put up without being cleaned? Prodigious!!! I know not what notions of cleanliness these persons imagine Medical Gentlemen possess, but for my own part, (in Enema cases for instance) if the operator were to put his instrument up "*sans ceremonie*" I should certainly expect to see him pocket the rectum pipe with an equal indifference.

Another equally untenable objection has been raised to the instrument, in consequence of a trifling attention being necessary as to position in withdrawing the contents of the stomach. In a former notice of this subject, I happened to say that the degree of inclination at which the Syringe should be held in this part of the operation, should make an angle of 45°; this remark has been a good handle for our interested hypercritics to turn the subject to my disadvantage. They have asserted that my Syringe cannot be used except it be held in *one particular* position, to discover which they modestly and humbly submit, would, in most cases, be an insurmountable attempt, from the want of mathematical knowledge in taking angles!!! Now the truth of the matter is simply this, that the position which I recommended (as shewn by the drawing page 8,) is really one to which *convenience* alone would involuntarily conduct the operator's hand if he had never been told a word about it. Let the reader imagine that he *stands* with the Syringe in his hand, fixed to the extremity of the Œsophagus tube projecting from the mouth of the patient, who is *seated* or *laying* before him, and the position of the Syringe he will find corresponds with that represented in the cut, *fig*. 2. When I alluded to the propriety of holding it at an angle of 45 degrees, I referred to the mathematical principles of the instrument, the valves of which lay directly on their bearings at this point of the scale; but I by no means intended to say (nor could it be so understood, except by wilful misinterpretation) that no other position could be effective; it is just otherwise; for the valve of the lateral branch of the Syringe covers its bearing, if the instrument be inclined to a level with the horizon, and it will sustain its proper seat upon the aperture during a *lateral* motion of half a circle. It resolves itself at last to this, that by the simple rule of keeping the lateral branch of the Syringe *upwards*, the instrument will act perfectly, and cannot fail.

My opponents have endeavoured also to depreciate the value of the instrument by asserting that much valuable time is lost in shifting the tubes during the operations upon the stomach. Had such a complaint proceeded from a medical practitioner, or any one who had actually performed or witnessed the operation, it would, indeed, be sufficient to "make the judicious grieve;" but coming, as it does, from persons incompetent to give an opinion, (the truth of which would be suspected even if they were) it can be viewed only as the dicta of "envy, hatred, and malice, and all uncharitableness:" that time is lost nothing can be more untrue, as will appear by the testimonials which I have presently to submit, of which any person may readily satisfy himself by a trial.

The size of the Syringe has been supposed by some persons to be too small, and they have imagined that with a larger instrument they could propel fluids with more power; but it is just the reverse, it being an acknowledged law in mechanics, that as you enlarge volume you increase resistance, and as you encrease resistance you diminish power. The force, then, with which fluids may be propelled with my Syringe, is four times greater than it would be if the instrument were twice its present size, and as that even, would not be half so large as other Syringes sold for this purpose, its power when compared with them is (to say the least) as 16 to 1, and with some infinitely more. The bulk of the fluid contained in the instrument is so small, that the force necessary to propel it, scarcely requires the efforts of an infant; but the effects of these efforts, multiplied by repetition, increase to an almost infinite ratio, and at length present an overwhelming force, capable of bearing down all opposition, and overcoming all natural restraints. To try the power of the syringe, I fixed the injecting pipe firmly into the rectum of an Animal that had been recently killed, and proceeded to pump into the bowels a large quantity of water, and I continued the operation with the same ease and freedom, until the intestinal canal, stretched beyond its tone, burst with the distending force.

Notwithstanding the smallness of the instrument, it is capable of injecting a larger quantity of fluid in a given time than is requisite in any case, particularly in Enema practice, where it is necessary to move the piston slowly, and allow a little time between each stroke.

My avocations in life have led me through all classes of society, and, amidst the variety of my duties, I have been honoured most particularly with the patronage (and I trust it will not be deemed presuming, if, in the gratitude and warmth of my feelings, I add, the *friendship* also) of the Medical Gentlemen in the counties of Kent and Sussex. During one of my occupations of this nature, with that respectable and amiable man, Mr. Newington, Surgeon, of Goudhurst, (in the year 1819) I learned that himself, and Dr. Wilmot, of Hastings, had recently lost a patient (whom they had been conjointly attending) with obstruction of the bowels. I ventured to enquire of these gentlemen, if there was no apparatus by which mechanical distension might be effected in these cases; they replied, that surgeons possessed no instrument by which a sufficient accumulation of fluid with an efficient power, could be properly directed. Convinced, as I was, from hydraulic principles, that both these objects could be easily effected, I instantly turned my attention to the subject, and in the course of the following year, I perfected my Injecting Syringe, for which I obtained a Patent in the month of August, 1820. By order of Sir William Blizzard, I submitted the Instrument to the inspection of the Court of Examiners, at the Royal College of Surgeons, who highly approved of it. Mr. Abernethy, in particular, was pleased to express his approbation of the principle upon which it was constructed. During the year 1821, most of the surgeons of this part of the country had possessed themselves of the Instrument, which having given them the most satisfactory results, they very liberally and *unsolicited*, gave the following testimony of its utility:

( COPY.* )

"*We, the undersigned, Professional Men, strongly recommend the use of the* PATENT INJECTING MACHINE, *Invented by* MR. JOHN READ, *as being the most efficient Instrument for the purpose of removing Obstructions in the Bowels; and declare that we have had, by experience, proofs of the most decided advantage it has over every other Instrument within our knowledge, invented for the same purpose.*"

| | | |
|---|---|---|
| " Robert Montague Wilmot, M. D. | | Hastings. |
| Robert Watts, M. D. | | Cranbrook. |
| William Duke. | Surgeon, | Hastings. |
| Thomas B. Satterley, | Do. | Do. |
| George Taylor, | Do. | Do. |
| James Duttan, | Do. | Do. |
| Robert Ranking, | Do. | Do. |
| Charles Stephen Crouch, | Do. | Do. |
| Robert Watts, | Do. | Battle. |
| James Watts, | Do. | Do. |
| Stephen Monkton, | Do. | Brenchley. |
| Jonathan Monkton, | Do. | Do. |
| Samuel Newington, | Do. | Goudhurst. |
| Charles Newington, | Do. | Ticehurst. |
| Edward Morris, | Do. | Tunbridge. |
| Richard Thompson, | Do. | Rochester. |
| Avery Roberts, | Do. | Lewes. |
| Henry Verral, | Do. | Do. |
| John Vine, | Do. | East Peckham." |

* Were I desirous to increase the List, I might obtain the signatures of five hundred practitioners, who have more lately satisfied themselves

In corroboration of the good effects of this instrument in obstructions of the bowels, I shall take leave to extract the following remarks from some of the most respectable medical publications of the present time.

" Dr. Chisholm has related a case of obstinate constipation of the bowels, relieved by *Read's Injecting Machine* after various other means had failed. The obstruction had existed three or four days before Dr. Chisholm saw the patient with Mr. Beet, Surgeon, of Ashford. When seen by Dr. Chisholm, the patient's extremities were cold, and stercoraceous vomiting had come on. A tepid solution of yellow soap was prepared, and more than *a wash-hand basin full* was gradually but perseveringly thrown up by means of the instrument above mentioned, and prevented from returning by napkins pressed to the anus. *The patient's belly now resembled a drum.* When the injection was allowed to come away, the spectators had the gratification to find it mixed with fœces. Shortly after this, the patient passed flatus and stools, and all the bad symptoms quickly vanished. "'I have had many other cases'" says Dr. Chisholm, "'where Read's Machine was of infinite service, and I think every medical practitioner should have one in his possession.'"—*(Med. Repos. No.* 1, *New Series, Page* 944.*)*

A recent Medical Author, under the article costiveness, makes the following remark : " But the use of clysters is in every way preferable to purgative medicines, and those who are costive should provide themselves with " Read's Patent Syringe," and administer a pint of the domestic enema every day at a certain hour, until the bowels act without." The

---

of the utility of the Instrument, but I prefer this document in its original state, as a *voluntary* testimonial at a time when the Instrument was little known, and had nothing but its simple merits to recommend it.

same author treating upon Iliac Passion remarks that "a copious injection of six or eight quarts of warm water, or gruel, will be the most likely means of removing the obstruction, restoring the bowels to their proper situation, and of softening and bringing away those hardened motions, which accumulate in the bowels and occasion the complaint. For this purpose (as well as for the injection of tobacco smoke,) *Read's Patent Syringe* is preferable to all other instruments, and should be in the possession of every family."

I am informed by some Medical Gentlemen who have used it, that in violent cases of *Menorrhagia,* they have been able to check the disease more effectually by an alum injection thrown by the force which the Patent Syringe affords, than by any other means.

Mr. Scott in his "sketch of the utility of Enemas," makes the following remarks upon the effects of costiveness.

"To obviate these complaints, recourse is generally had to the use of purgative medicines, which most frequently aggravate the mischief and occasion new disorders. In this species of practice, the French and other continental nations have long pursued a much more rational and beneficial mode of treatment; instead of swallowing a host of drastic drugs which nauseate the stomach, irritate the bowels, and disorder and debilitate the constitution at large, they apply a simple remedy at once to the offending organ in the form of clyster, which if properly prepared and administered, softens and dissolves the contents of the bowels, removes obstructions by the mechanical distension it produces, and by its gentle stimulus restores a healthy tone and action, without inconvenience, debility, or pain.

"To give, however, this desirable plan its proper efficacy, an instrument was wanted, not only adapted to domestic use, but which could meet all the exigencies of those severe cases of obstruction that often baffle medical skill, and terminate fatally. For the first purpose, it was necessary that the machine should be so constructed that an invalid should be able to use it *without assistance:* and for the second, that it should be capable of transmitting *any quantity* of fluid desired, with a power equal to the resistance it might experience.

" This has lately been effected by the invention of a small Syringe or Pump, by an ingenious person, named Read, which is more suitable to this operation than any other instrument hitherto used. The Cylinder of this Syringe is not more than three quarters of an inch in diameter, and three inches and a half in length, and receives about an ounce of fluid, which is admitted at the extremity, and discharged through a small branch at the side attached to a long flexible tube that conveys it to the bowels. Notwithstanding the small size of this instrument, a large quantity of fluid may be injected in a very short space of time; in fact, it can be made to pass with a velocity not requisite in any case to which it may be applied, *viz.* at the rate of three quarts per minute. The French and other Clyster Syringes (containing a pint or more) are much too large to be either convenient or efficacious; in the first place, if there be any obstruction in the intestinal canal, or the bowels oppose the passage of the injection by any degree of reaction (which they usually do) the force necessary to propel so large a column of fluid requires the arm of a Sampson or a Hercules; and secondly, the clumsy size of these instruments renders their use so awkward, that the patient is often much hurt by the attempts to effect the operation. Besides this, if a large quantity of fluid be necessary (as in cases of introsusception, obstinate constipation, &c. where several pints or even quarts are often thrown up) the operation is unavoidably suspended as often as the instrument requires to be recharged, and this, perhaps, several times successively. There are, also, serious objections to Machel's canister apparatus, the fluid contents of which are forced into the bowels by the agency of condensed air. One of the evils of this instrument is, that part of the confined atmosphere rushes through the liquid injection and passes into the bowels along with it, occasioning, of course, mischievous and hazardous consequences; and again, as the injection is forced out by the expansive action of the compressed air within the canister, consequently, the propulsive power *lessens* as the operation *proceeds,* which is directly the reverse of what ought to happen, for with an accumulating resistance and volume anteriorly, the *vis a tergo* ought to be, of course, proportionally increased.

"None of these objections appertain to Read's Syringe, the action of which is so easy that it may be worked with a finger and thumb, whilst its power is so great that all resistance yields to it without any increased efforts."

I shall add one more testimonial in favor of my instrument as an Enema Syringe, which is a letter I have just received from a surgeon, whose talents and experience are held in high estimation in the counties of Kent and Sussex.

*Goudhurst, March* 20, 1826.

Mr. Read,

After five years observation, I am so convinced of the great utility of your Enema Syringe, that I think an acknowledgement of this conviction is due to you; and so perfectly am I satisfied of its superiority above any other apparatus of its kind, that I should be wanting in justice to the profession, the public and yourself, if I longer neglected to recommend it strongly to their attention.

I am, your's, &c. &c.

Saml. P. Newington.

As an apparatus for removing the contents of the stomach, my Instrument, as most persons are aware, was first ushered into notice by Sir Astley Cooper, the particulars of which have long been before the public. I shall, therefore, only recapitulate so much of what has already been reported of the worthy baronet's, remarks, as tend to contradict an idle assertion, that the Syringe was incapable of removing *mineral* poisons.

In addressing the class who had recently witnessed Mr. Scott's successful experiment upon Mr. Jukes, in the theatre of St. Thomas's Hospital, and that of himself upon the dog, at Guy's Hospital, Sir Astley thus proceeds:

"I certainly think, however, after the experiment which you had an opportunity of witnessing in this theatre, and that of the dog in the other hospital, that the instrument for evacuating the stomach affords the best means of saving persons, who would otherwise perish under the influence of opium. I mentioned to you on a former occasion the case of the young lady who had taken opium, in which every means which I could employ for the purpose of producing vomiting proved completely unavailing. When the œsophagus has lost its functions, which it soon does from the influence of opium, no stimulating substances will produce the least effect upon it. I sat hour after hour, by the side of this young lady, watching her progress to dissolution, without being in the least able to prevent it. If, however I had been acquainted with the instrument which has been since invented, I should have used it with the probability of success. This instrument enables us not merely to remove the poison from the stomach, but to throw in water in considerable quantities, and to introduce stimulating remedies after the opium is removed, for the purpose of restoring the functions of the Nervous system; and this in cases where emetics cannot be even swallowed. I certainly do expect the happiest results in such cases from the invention of this instrument. The man who first suggested such an idea deserves well of his country, and they who oppose it until the instrument has been fairly tried and found useless, must be destitute of understanding. Persons who object to a proposition merely because it is new, or who endeavour to detract from the merit of the man who first gives efficacy to a new idea by demonstrating its usefulness and applicability, are foolish, unmanly, envious, and illiberal objectors; they are unworthy of the designation either of professional men, or of gentlemen."—LANCET, Vol. III. No. 6, page 174.

In speaking of the treatment of poisoning by the oxymuriate of quicksilver, Sir Astley remarks;

"It may appear that I am disposed to think too well of the instrument to which I before adverted, when I state that I believe the Syringe may also be successfully employed for the purpose of removing the oxymuriate of mercury from the stomach. I should certainly prefer it to any other means; but instead of using simple water, I should throw in a quantity of soap and water, then withdraw it; I should repeat this operation until the stomach was entirely cleansed. It has been suggested that although this instrument may be used with success for the purpose of removing the vegetable poisons from the stomach, yet it would not succeed in cases of poison by arsenic or corrosive sublimate. *This I do not believe.* With respect to arsenic, I am aware that if it were taken in a solid form, and a considerable portion had fallen on the stomach it would be impossible to remove it; but as it is usually taken, in powder, I think the instrument is very capable of removing it, because it will be for a considerable time at least kept in solution by the mucus which is thrown from the surface of the stomach, and in this state it may be removed. At all events this deserves a trial."—LANCET, Vol. III. No. 6, page 177.

Several cases in point have since occurred to verify Sir Astley Cooper's opinion, one of which is related by Mr. Campbell, a Surgeon of Pimlico, of a young female, who swallowed, by mistake, a quantity of corrosive sublimate, but instantly discovering the error, a quantity of the white of eggs were administered to decompose the oxymuriate, and the tube being passed into the stomach, the contents were extracted by the Syringe, and the patient experienced no ill effects.—*See the Morning Chronicle of Friday, September* 17, 1824.

A case of this nature occurred also soon after the above. A young woman in a state of pregnancy, endeavoured to destroy herself by taking an ounce of Sugar of Lead. Copious vomiting was produced by very powerful emetics, but the pain of the stomach remained extremely severe. Under these circumstances, Mr. Scott, assisted by Mr. Iliff, of the West London Dispensary, and Mr. Mason, Surgeon, of Newington, injected the stomach with warm water by the Patent Syringe, the force of which dislodged the poison adhering to the inner coat of this organ, and effectually removed the pain as soon as the fluid was withdrawn. In this case also the Syringe, as an enema apparatus, proved most essentially serviceable; for a portion of the lead having passed into the bowels, constipation and colic succeeded, which were removed by an injection of Epsom salts in warm water; *six* pints of which were thrown up.

Another important and successful operation upon the human stomach, where metalic poison had been taken, has been lately performed by Mr. Roberts, Surgeon, of Brighton, of whose report the following is an abstract.

" A young man having swallowed a teaspoonful of arsenic, myself and Partner (Mr. Blaker) were called to him. We took with us that excellent Instrument, READ'S STOMACH SYRINGE, and although he made every exertion to prevent us, we found the introduction and application of the Tube and Syringe extremely easy. The whole contents of the Stomach were well washed out, and to make the removal of the arsenic certain, two gallons of water were introduced and withdrawn: the young man, in a few days, was able to resume his occupation.— An attempt has been made to prove that much valuable time is lost through the peculiar mechanism of this Syringe, but I must observe, that if we can remove arsenic from the Stomach with great facility and with sufficient expedition to save life after the Poison has been swallowed nearly half an hour, *there can be no well grounded objection* brought against the Apparatus.

AVERY ROBERTS, Surgeon."

"15, Jerman Place, Brighton,
Dec. 20, 1825."

The above is also a triumphant reply to the cavilling sophistry of those who artfully insinuate that time is lost in the use of my Syringe' and further to confute the malignant aspersion, I shall add the following testimony, obligingly given me by Mr. Scott, who is eminently calculated to give an uncontrovertible opinion upon the merits of my Syringe, not only from his having been the first person to operate upon the human subject, but from the long and unwearied attention he has devoted to the mechanical construction and philosophical improvement of a proper apparatus for the purpose; to which may be added his experience in its practical application, more cases having fallen into his hands than has occurred to any other professional gentleman in England.

"I have not seen any instrument so eligible for removing poisons from the stomach as "READ'S PATENT SYRINGE."

The simple construction of the pump affords such a ready and unembarrassed action, that the stomach may be cleansed in the space of three or four minutes.

J. SCOTT,"

*London, March* 25, 1826.

The following remarks are to be seen in Dr. Johnson's Quarterly Review.

"For many months past we have been in the habit of employing Mr. Read's Patent Injecting Apparatus, which is so small as to be carried in the waistcoat pocket, and so powerful as to throw fluids to a great distance. The object of our present notice, however, is to inform our readers that Mr. Read has adapted to the Instrument, a flexible elastic tube, most admirably calculated for throwing fluids into the stomach, and then extracting them in cases of poisoning. We have attentively examined the instrument, and we know it is approved of by Sir A. Cooper, and some of the first Surgeons of the Metropolis; we think it of so much importance, that we seriously recommend it to every private practitioner." Vol. 4, No. 15, page 742, of the Medico-Chirurgical Review.

As a concluding testimony in favour of my apparatus I beg leave to subjoin the following letter, which is authenticated by the signature of the Chairman of the Committee of Governors of the Northampton Infirmary, a favour altogether unsolicited and unexpected.

*General Infirmary, Northampton.*

SIR,

I am directed by the Committee of Governors of this Infirmary, to convey to you their approbation of your Instrument for extracting poisons from the stomach, and to give you the details of a case in which it was used with complete success.

A boy, nine years of age, was discovered at eight o'clock in the morning of the 12th ult. in nearly a lifeless state. On investigation it was ascertained that he had taken, by mistake, a solution of opium, three hours before. He was lying in a deep stupor, his respiration very slow, and accompanied with a convulsive catching; his feet, hands, and face livid, and no pulse to be felt at the wrist. He was immediately roused up, and violently shaken, when he uttered a few incoherent cries. A quart of warm water was instantly injected into the stomach by means of your Syringe, and then withdrawn; the fluid was brown, and the smell of opium plainly perceptible. Another quantity of water was then thrown in, and withdrawn; it returned colorless and without any smell.

The boy was now moved continually about for some time, and his senses gradually returned. As soon as he could swallow, he was made to drink two ounces of Ipecacuanha Wine, with a drachm of Sulphate of Zinc, dissolved in half a pint of warm water. This not operating, in twenty minutes a second dose was given as strong as the first, and in ten minutes afterwards the boy showed a disposition to vomit; this was effectually excited by injecting a hand-basin full of warm water, by which I made sure that his stomach should be completely washed of any remains of the poison. After the vomiting was over, he was kept in motion for three or four hours, taking at intervals a strong decoction of coffee: by the afternoon of the same day I had the pleasure of finding him perfectly well.

It is almost unnecessary to observe, that as the opium had been swallowed three hours, (and that too upon an empty stomach,) no emetic medicine would have operated until the poison was withdrawn; the fibres of the stomach being rendered perfectly inert by the stupefactive effect of the drug; indeed he had totally lost the power of swallowing; it is therefore pretty evident, that the boy's life would not have been saved, but for the very useful Instrument of which you have the merit of being the inventor.

I am, Sir,

With much respect,

Your obedient Servant,

CHARLES WITT,

*House Surgeon.*

Approved,

C. BOUVERIE,

*Chairman of the Committee.*

To Mr. READ.

In the preceeding remarks, I have been actuated by no wish to depreciate the labour of other persons, desirous only of correcting misrepresentations insidiously made to disparage my own. Nor have I out of vanity, here adduced the respectable professional testimonials with which I have been honoured, but as a vindication, much more powerful than any that my own feeble attempts could effect, and for which (and to the profession in general from whom I have received many valuable suggestions and the utmost urbanity) I most respectfully beg to offer my humble and very grateful acknowledgements.

30, *Bridge House Place,*
*Newington Causeway, London.*
*April* 1, 1826.

JOHN READ.

---

*** **The Instrument is Manufactured and Sold by the Patentee, to whom it is requested Orders may be addressed as above.**

// READ'S

**NEW IMPROVED PATENT SYRINGE,**

*FOR DOMESTIC & HORTICULTURAL PURPOSES.*

THE Public are respectfully solicited to the Inspection of this Instrument, which for its convenience and utility in Domestic and Horticultural Purposes, claims universal regard. For watering Pines and all other plants in Conservatories and Hot-houses; and for the destruction of Insects upon trees in Forcing-houses or on Walls, it far exceeds the Barrow-engine in the facility of its application. The Horticultural Society of London, to mark their approbation of it, have been pleased to honour the Patentee by conferring upon him their Silver Medal for the Invention. It has of late been much used for washing the Windows of Houses and Carriages, and is found to be a most effective apparatus for Fumigating Trees and Hot-houses.

This Instrument also, in case of need, is an excellent Fire-engine, as from its portability it can be applied, upon the first breaking out of a Fire, when no sort of assistance could be derived from the Engines of the Insurance Companies, and its utility in this way having been proved by actual experience, most of the Fire Offices have prepared themselves with it, and it is now, very properly finding its way into Private Families, as a safeguard against the destructive and hazardous effects of Fire.

### *Section of the Horticultural Syringe.*

*a.*—Cap for destroying Insects on Wall-trees and Plants.

*b.*—Cap for Watering Forcing-houses and trees in Blossom.

*c.*—Cap for Extinguishing Fires.

*d.*—Cap for the Tobacco Fumigation.

### *EXPLANATION OF ITS USE.*

The Cap *a* is to be screwed on when the Syringe is used for washing away Insects from Peach, Nectarine, and Apricot Trees. Set a pot of water near the stem of the tree, and having charged the Syringe, throw the shower between the tree and the wall, directing it against the *back* surface of the leaves, where the insects are placed, by which mode, the fluid effectually and speedily sweeps off both the insects and their eggs and larvæ, and thus prevents a succession of these injurious animalculæ. The Barrow Engine can only be brought to play upon the *front* of fruit trees, and dislodges, therefore, the insects but very imperfectly, without removing, in the least, their eggs, that stick upon the under surface of the leaf. This Cap is also used for watering Pines.

The Cap *b*, has smaller perforations than the above, and as it throws the fluid in a light and gentle moisture, almost like a dew-fall, is particularly eligible for sprinkling Forcing Houses of all descriptions, and Trees in bloom, and not only clears the latter of insects, but deposits the water in such a gentle manner upon the leaves, that, if it be applied at night, preserves the plant moist until the next morning, materially tends to its nourishment and health, and prevents the formation of animalculæ, which breed rapidly in the *dry* but perish by moisture. The Practical Gardener is aware of this, and takes care, during warm weather, to supply his trees with moisture while their buds are forming and before the blossom expands. This Cap is used also for washing the leaves of trees, plants, and vegetables when frost-nipped in the cold nights that often prevail during the spring; it should of course be done before sun-rise.

The Cap *c* is used for extinguishing fire and for washing the coarser sorts of trees, as Pears, Plumbs, Cherries, &c. against walls, and for general watering in lieu of the Barrow Engine, and in this way can be applied more efficaciously than the latter as it may be brought into immediate contact with the plant, or applied in any direction that may be desirable, which the Barrow Engine cannot, on account of the impracticability of bringing it over the beds.

By the application of the Syringe there is no useless expenditure of water, and it is generally found that two, or at most three charges is sufficient for a large tree.

The Fumigating canister-*d*, is used in the following manner. Having fitted the brass tube to the side opening, unscrew the top, take out the perforated plunger, and put about an ounce (or as much as is desired) of tobacco (or tobacco paper, as it is called) into the canister, replace the plunger and allow it to sink upon the tobacco with its own weight only, and having put on the top, screw it to the Syringe, and next apply a piece of lighted paper to the nozzle of the canister when one or two strokes of the piston sufficiently lights the tobacco, the fumes of which instantly pass in a copious dense stream from the extremity of the side tube, and may thus be readily conveyed to any plant, or even to any part of a plant. When applied to beds of roses or to plants under walls, the operation is greatly facilitated by throwing a piece of canvas over the bed, or hanging it against the wall so as to cover the trees. The canister is not liable to become choaked as the Fumigating Bellows are, but continues to act freely until the tobacco is entirely consumed.

The Patentee, after an active and extensive experience of Forty Years in Practical Gardening, humbly offers the above explanation of the uses of his Garden Syringe to the attention of young Horticulturists, who may not despise a few simple but useful hints.

Neither of the above Instruments are genuine except Stamped with the Royal Arms and Patentee's Name.

### Veterinary Practice.

" A righteous man regardeth the life of his beast."

PROVERBS, chap. xii. v. 10.

ANIMALS, as well as man, are liable to accidents and disorders that demand the aid of medical surgery; and among these, the occurrence of constipation and obstruction of the bowels, and of the fatal effects of excessive abdominal distension, from an undue quantity of improper food, frequently brings a most useful and highly-valued animal into a situation of the utmost danger. Examples of the former are constantly experienced with horses and dogs. The former possess a tendency to costiveness, from the dry nature of the food with which he is supplied, under the general routine practice of feeding; and he is rendered still more susceptible of this state, and consequently of obstruction and even inflammation, by protracted and heavy labour, and by neglect or improper management after severe exercise. It is also a well-ascertained fact, that the sports of the field induce a costive state of the bowels of dogs, that often reduce the animal's condition and health, and not unfrequently destroy his life. The attention of sportsmen and gentlemen cannot, therefore, be too seriously drawn to this subject; and I respectfully beg to solicit their consideration of an instrument by which the lives of many valuable animals have been saved, when every other means had failed. By means of the apparatus represented by fig. 1. of the following plate, enemas may be easily administered either to horses or dogs; and the instrument is such as to admit of any quantity being injected that may be considered applicable to the size of the animal, and to the nature of the case.

Fig. 1.—*a*. Enema Tube for Horses.

*b*. Ditto do. Dogs.

*c*. Vessel containing the Injecting Fluid.

Fig. 2.—Injecting Apparatus for Hoven or Blown Cattle.
*d*. The Œsophagus Tube for Bullocks.
*e*. Ditto do. Sheep.

The plate (fig. 1.) represents the action of the instrument: the tube being screwed to the side branch of the syringe, and the pipe introduced into the bowels, the extremity of the syringe is held in the fluid to be injected, (which is put into a pail, or other convenient vessel) and the piston being put into action the clyster passes freely into the intestines. The facility afforded by this instrument of throwing fluids into the bowels of animals was demonstrated by an experiment performed at Charlton Mews, before Mr. Goodwin, his Majesty's Veterinary Surgeon, in which I injected a clyster of three gallons in two minutes.

The next consideration, as to the applicability of the instrument, is to the cases of hoven (or blown) cattle. The frequency of this occurrence to bullocks and sheep * from over-gorging themselves with potatoes, turnips, flax-seed, ground meal, green clover, or any moist or succulent food, is unfortunately well known to the agriculturist and every person practically engaged in the breed and management of stock: and it has been often experienced, that the means generally resorted to, in those cases, are, but too frequently, ineffective. The failure may be accounted for by observing how inadequate either the puncture in the loin, or the introduction of the tube is to the evacuation of the offending matter; if this were merely gas, either of the above means would probably liberate it: but it should be known that the stomach is filled by a fermenting pultaceous mixture of solids, fluids, and gas, that cannot be discharged in the manner of gas simply. The Patent Syringe before described, is found to be as exactly applicable for this as for any other purpose; and I have prepared tubes to be fixed to it, either for sheep or bullocks, see plate *d. e.* Fig. 2. shews the operation of extracting the contents of the stomach of a blown bullock—the tube is passed into the stomach, and the syringe being fixed to it, and put into action, the offending matter is discharged at the side opening.

---

* Horses have been destroyed by eating largely of wheat, obtained by their breaking into a barn where it had lately been thrashed out.

---

*Sold by the Patentee,* 30, *Bridge House-place, Newington Causeway.*

---

GLENDINNING, PRINTER, 25, HATTON GARDEN, LONDON.

## Figures and Tables

**Fig. 1. Fig. 2. f1:**
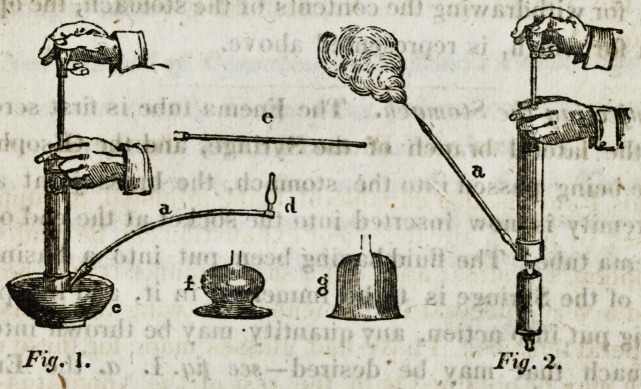


**Fig. 2. Fig. 1. f2:**
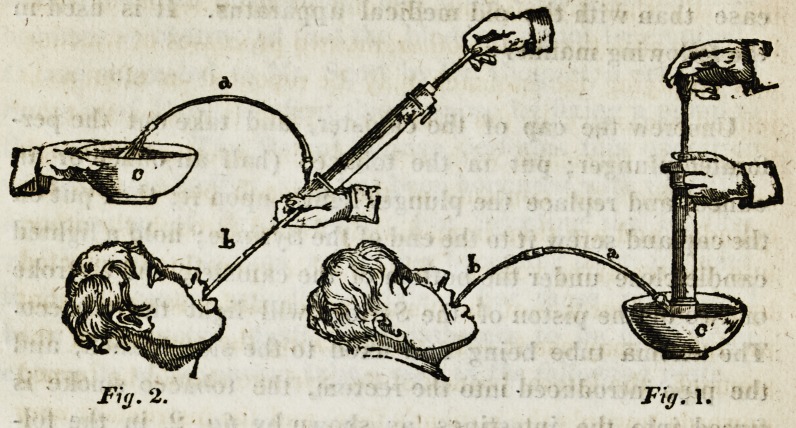


**a f3:**
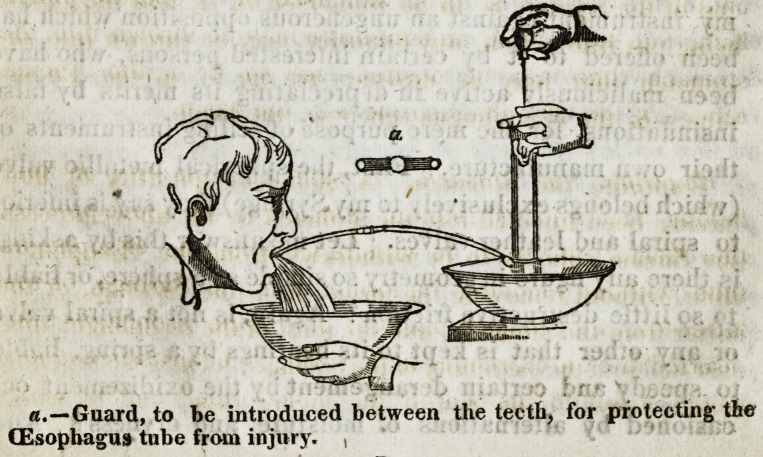


**d c b a f4:**
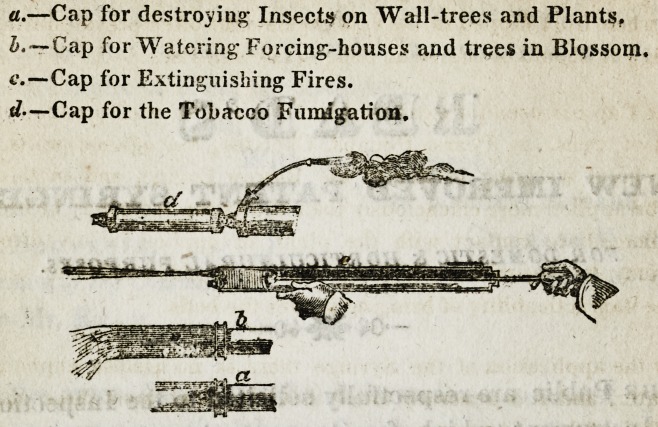


**Fig. 2. Fig. 1. f5:**